# Mild-to-Moderate Traumatic Brain Injury: A Review with Focus on the Visual System

**DOI:** 10.3390/neurolint14020038

**Published:** 2022-05-30

**Authors:** Steven H. Rauchman, Jacqueline Albert, Aaron Pinkhasov, Allison B. Reiss

**Affiliations:** 1 The Fresno Institute of Neuroscience, Fresno, CA 93730, USA; 2 Department of Medicine, Biomedical Research Institute, NYU Long Island School of Medicine, Mineola, NY 11501, USA; jacqueline.albert@duke.edu (J.A.); allison.reiss@nyulangone.org (A.B.R.); 3 Department of Psychiatry, NYU Long Island School of Medicine, Mineola, NY 11501, USA; aron.pinkhasov@nyulangone.org

**Keywords:** head injury, prognosis, traumatic brain injury, vision, retina

## Abstract

Traumatic Brain Injury (TBI) is a major global public health problem. Neurological damage from TBI may be mild, moderate, or severe and occurs both immediately at the time of impact (primary injury) and continues to evolve afterwards (secondary injury). In mild (m)TBI, common symptoms are headaches, dizziness and fatigue. Visual impairment is especially prevalent. Insomnia, attentional deficits and memory problems often occur. Neuroimaging methods for the management of TBI include computed tomography and magnetic resonance imaging. The location and the extent of injuries determine the motor and/or sensory deficits that result. Parietal lobe damage can lead to deficits in sensorimotor function, memory, and attention span. The processing of visual information may be disrupted, with consequences such as poor hand-eye coordination and balance. TBI may cause lesions in the occipital or parietal lobe that leave the TBI patient with incomplete homonymous hemianopia. Overall, TBI can interfere with everyday life by compromising the ability to work, sleep, drive, read, communicate and perform numerous activities previously taken for granted. Treatment and rehabilitation options available to TBI sufferers are inadequate and there is a pressing need for new ways to help these patients to optimize their functioning and maintain productivity and participation in life activities, family and community.

## 1. Introduction

Traumatic brain injury (TBI) is an imminent global health challenge and the primary cause of trauma-related long-term or permanent disability worldwide [1]. In 2019, the CDC reported 61,000 deaths related to TBI [2]. With incidence rates of 50 million cases per year, TBI may be categorized as mild, moderate or severe [3]. Mild-to-moderate TBI accounts for about 90% of all TBIs while approximately 80% of TBI cases in the United States are classified as mild (mTBI) [4,5]. Severity and morbidity are disproportionately high among lower- and middle-income countries and the estimated global economic cost is 400 billion USD annually [6,7,8].

The assumption that mTBI has little consequence has been debunked as it may result in neurological symptoms and cognitive impairment with tangible impacts on quality of life and substantial demands on health services [9,10].

## 2. Methods

An extensive literature review was performed using PubMed and Google with regard to the topics “mild or moderate traumatic brain injury”, “concussion” and “traumatic brain injury, eye, and vision”. The search terms included “traumatic brain injury”, “concussion”, “visual pathways AND traumatic brain injury”, “parietal lobe AND traumatic brain injury”, “traumatic brain injury AND treatment”, “traumatic brain injury AND medications”, “traumatic brain injury AND rehabilitation”, “traumatic brain injury AND quadrantanopia”, and “traumatic brain injury AND imaging”. Studies available in English from 1989 onward were included. This yielded approximately 1000 manuscripts from which we narrowed the scope by looking at human studies (observational, randomized, prospective and retrospective) as well as meta-analysis papers and reviews of each key topic area. We excluded studies that focused primarily on severe TBI and hospitalization. The final number of papers reviewed after applying these selection criteria was 200.

## 3. Basics of TBI

### 3.1. Detemining Severity

Manifestations of TBI occur after an external mechanical force is sustained by the patient in the area of the head, neck and/or face leading to a primary injury characterized by neuronal impairment [11]. The most commonly observed clinical features of mTBI are headache, dizziness, nausea and poor concentration [12]. More severe injuries can lead to aphasia, seizures, amnesia, behavioral abnormalities and, in the worst cases, coma [13]. These may manifest within seconds to minutes following TBI. The most widely used clinical assessment of TBI is the Glasgow Coma Scale (GCS) which classifies TBI as mild (14–15), moderate (9–13), or severe (3–8) [14,15]. The GCS is comprised of the eye opening, verbal, and motor subscales, which are combined to give a total GCS score. The GCS system has several shortcomings due to its ambiguity in diagnosing mild and moderate TBI. For instance, there is still persistent disagreement on whether a GCS of 13 should be treated as mild or moderate TBI [16]. Other notable challenges to the accurate diagnosis of TBI stem from inter-rater variability and a plurality of etiologies as well as a general disagreement in the literature over which of the many possible TBI symptoms should be used to determine disease severity [17]. Even after an accurate diagnosis, different patients with similar diagnoses on the GCS can present a multitude of outcomes due to underlying genetic factors, the type of injury, and the severity of the secondary injury that occurs due to the initiation of damaging biochemical cascades by the primary injury [18].

### 3.2. Initial Treatment

A large concern of TBI treatment relies on immediate therapeutic intervention to prevent secondary injury, as the primary injury cannot be undone. Secondary injury can present minutes to days after the initial insult due to neuro-inflammation, changes in cranial blood pressure, and the disruption of neurological homeostasis resulting in neuronal cell damage, apoptosis and death [19]. There is no pharmacological medication with proven efficacy for human TBI. Current treatments aim to prevent hypoxia, hypercapnia and hypotension and regulate cerebral perfusion pressure (CPP), ensuring that euvolemia is maintained and secondary injury is avoided [20,21].

### 3.3. Diagnostic Issues in mTBI

There is a lack of attention paid to mTBI since it is not regarded as an imminent medical emergency. This can result in the dismissal of patients from serious medical care and the failure to adequately characterize the severity of underlying neurological impairment. Additionally, many patients who have sustained mTBI injuries do not seek medical care, or are treated by healthcare providers lacking specific experience in this area. This is a pressing issue, since there exist a multitude of significant and persistent complications for mTBI patients such as impairments in cognition and motor function, psychiatric problems, and impaired neurological development in children [22]. Given that approximately 80–90% of TBIs are classified as mild, this neglects the lasting neurological implications of injuries for a large majority of TBI patients [23,24].

Public awareness surrounding the high incidence of mTBI has increased in recent years due to the publication of data surrounding high levels of mTBI complications in athletes and veterans, in particular [25]. Another subgroup with high rates of mTBI includes victims of abuse, who are a vulnerable and oftentimes neglected population. A greater emphasis on the treatment of mTBI and the management of its prolonged neurological consequences would have significant implications on quality of life for affected individuals [26,27].

## 4. Focal and Diffuse Injury

Traumatic brain injury results from either a blunt force directly striking the head in a closed or penetrating strike, or due to non-impact force. This initial strike results in a primary injury, which encompasses both direct brain damage caused by the sustained impact and the subsequent damage caused by the impairment of cerebral blood flow and alterations to homeostatic metabolism [28]. The type of primary injury sustained from the blunt or non-impact force usually fits into one of two broad categories: focal and diffuse injuries. Focal brain injury, often affecting the frontal and temporal lobes, results from the compression of brain tissue specifically at the site of impact due to collision forces acting on the skull and has clinical manifestations such as subdural and epidural hematoma and hemorrhagic contusions [29,30]. The temporal lobes are particularly vulnerable to the physical compression and vascular disruption that accompanies focal brain injury, perhaps because the bony covering is thinner relative to the bone over the frontal lobes. The frontal lobes also receive some cushioning from the air-filled sinuses. Since the temporal lobes harbor important memory-related structures, even mild contusions can lead to significant and enduring impairment [31]. Impacts to the frontal cortex can manifest as poor judgement and problem-solving abilities [32]. Focal TBI can disrupt the blood brain barrier (BBB), leading to cellular fluid extravasation into the extracellular space [33]. The cerebral blood flow may be altered, leading to hypo- or hyper-perfusion [28]. These homeostatic disruptions can cause brain tissue destruction, neuronal necrosis, and the formation of brain cavities due to glial cell reactivity [34]. Damage to the BBB is implicated in chronic inflammation after TBI, likely a result of microvasculopathy, which can lead to post-injury development of epilepsy and other neurological disorders [35].

**Figure 1:**
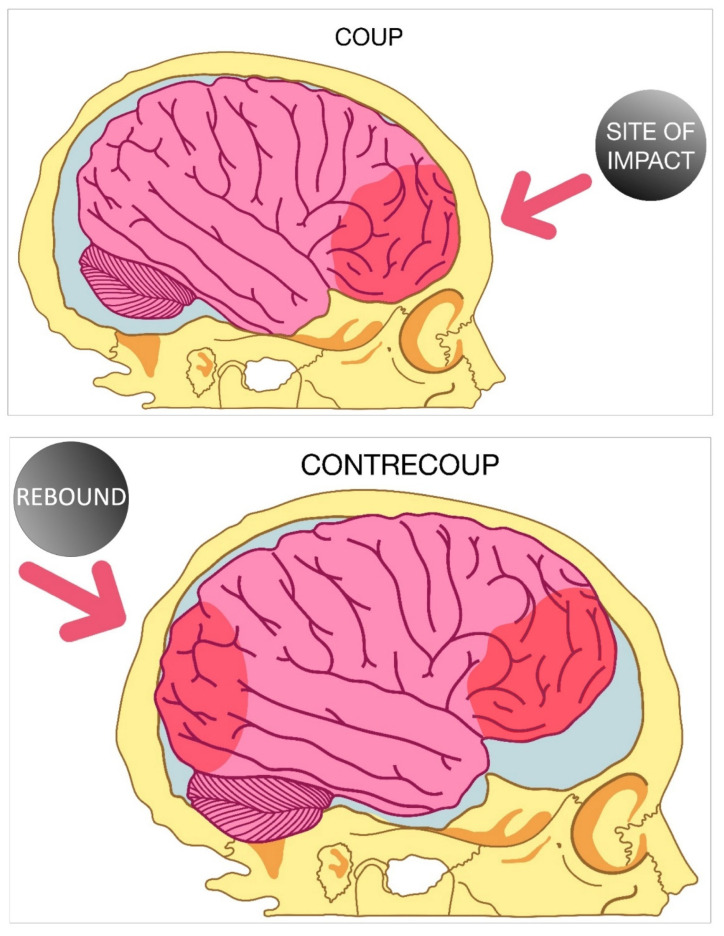
Coup contrecoup traumatic brain injury. The coup portion of the injury occurs when the movement of the head stops abruptly and the brain continues to move in the forward direction so that it hits the skull. The contrecoup portion further compounds the damage as the brain bounces off the skull and hits the side of the skull opposite the side of initial impact.

Contrecoup brain injury is a specific subset of focal traumatic brain injury in which the major cerebral contusions occur on the side opposite from the site of blunt force impact. Mechanistically, this phenomenon can be explained by visualizing the brain, which at rest is encased in the skull and floating in the cerebrospinal fluid (CSF). When the head rapidly accelerates, and then suddenly decelerates, the brain is displaced in the denser CSF in relation to the skull and collides with the internal skull in the contrecoup location [36,37]. This is seen when the brain collides with the skull and then rebounds in the opposite direction (coup-contrecoup), causing additional brain injury across from the location of blunt force impact (Figure 1). Coup-contrecoup injuries can lead to widespread damage due to the additional site of injured tissue at a remote location in the brain, resulting in a broadened array of symptoms in patients. This has a particularly strong effect on visual symptoms of TBI, as accommodatively-based visual symptoms such as trouble focusing eyes, visual fatigue, and blurred vision are highly correlated with coup-contrecoup injury [38].

Diffuse brain damage generally occurs after rapid acceleration-deceleration of the head, and is associated with disorders of consciousness related to axonal and vascular injury as well as brain swelling [14]. Diffuse damage is often detected via CT scans, and a recent influx of magnetic resonance imaging (MRI) data due to advancements in imaging technology have suggested a relationship between the presence of diffuse axonal injury (DAI) and worse outcomes of TBI [39]. The location of axonal shearing or sustained focal lesions in DAI heavily affects patient outcome, with common locations including the corona radiata, corpus callosum, internal capsule, brainstem, and thalamus [40]. Many cases of fatal DAI contain three specific hallmark structural features: focal lesions in the corpus callosum, focal lesions in the rostral brain stem and diffuse axonal damage [41]. These features are difficult to identify in living patients, complicating not only the diagnosis of DAI but also the ease of studying less severe cases of DAI that do not result in death.

## 5. Brain Imaging Techniques in TBI

The most widely used brain imaging technologies for the diagnosis of TBI include standard CT and MRI scans. Standard noncontrast CT scans are preferentially employed for rapid and comparatively low-cost imaging results, especially in cases of critical moderate and severe TBI where immediate medical intervention may be required [42]. Repeated CT imaging is controversial, but has shown promise in improving patient outcome in several studies [43,44]. However, CT scans present several concerns pertaining to ionizing radiation exposure in vulnerable age groups, such as children and pregnant women [45,46,47]. Performing noncontrast CT scans also has significant limitations in TBI prognosis, including inaccurately displaying the severity of early traumatic contusions, limitations for detecting changes in intracranial pressure and cerebral edema, and difficulty in identifying diffuse traumatic injury [48].

The predictive value in determining prognosis is comparable for MRI and CT scans, with the added advantage of MRI of an increased sensitivity in detecting small contusions and hemorrhagic injury to axons [49,50]. The drawbacks of MRI in comparison to standard CT imaging include high cost, lower accessibility of MRI machinery, and longer duration of time to obtain results. CT is also superior in detecting skull fractures and CSF leak [51]. The most common acute and chronic finding on CT or MRI of the brain is a normal exam. Thus, the routine neuroradiological investigation of head trauma often performed in the emergency department is insensitive to the structural abnormalities that suggest a patient has undergone TBI. CT imaging usually appears normal when investigating subacute TBI (more than 7 days and less than 3 months after the primary injury) or chronic TBI (3 or more months after the primary injury) [52].

Several classifications centering on CT readings have been developed for risk stratification and prediction of mortality of TBI patients. These include Marshall, Rotterdam, Stockholm, Helsinki and NeuroImaging Radiological Interpretation System (NIRIS) scores [53,54,55,56,57,58] (Table 1).

The use of single photon emission computed tomography (SPECT) to detect abnormalities in regional cerebral blood perfusion (rCBF) allows for high resolution and detection of small perfusion differences that may aid in predicting the likelihood of recovery [59,60]. SPECT is a functional brain imaging tool that uses a gamma-emitting radionuclide that can cross the BBB to show regions of abnormal blood flow [61,62]. Performing a SPECT scan is a minimally invasive method of assessing regional cerebral blood flow, therefore providing useful information on the relative activity levels of different regions of the brain to help detect pathologically significant brain perfusion patterns [63,64,65]. The utility of SPECT comes into play particularly in cases where structural abnormalities are not found on CT. Irregularities in brain perfusion can be seen immediately after mTBI and can identify regions of both hypoperfusion and hyperemia as well as other tissue dysfunction local to sites of brain lesions, indicating BBB disruption [66].

Abnormalities in rCBF are most easily detected in moderate and severe cases. The detected abnormalities in rCBF are most commonly located in the frontal or parietal lobe for patients with traumatic brain disorder [67]. In approximately 50% of SPECT scans of TBI patients, abnormalities in the occipital lobe (the visual cortex) are also detected. Abnormalities in rCBF that are localized in the visual cortex manifest clinically in cortical visual impairment. Therefore, it is relevant to consider the visual findings in TBI for patients with mTBI [68].

Sequential SPECT scans can be used to track the clinical evolution of a TBI patient throughout the duration of treatment [67,69]. SPECT can be used as a marker for improvement. Normalizing blood flow over time (studies months or years apart) usually indicates clinical improvement. If a new drug is developed that may improve the long-term outcome of TBI patients, it would be very useful to have serial SPECT scans to help prove the drug is actually working. Imaging of the brain is critical in making a correct neurologic diagnosis. The limitations of the immediate head CT in the emergency room have been previously described. SPECT is not indicated in every case, but merits addition to the arsenal of tests as a companion to CT for evaluation of TBI patients.

## 6. Visual Symptoms of TBI

A significant percentage of patients with mTBI report visual symptoms. Among the most common of these is photophobia, a form of light sensitivity in which light exposure causes eye and head pain [70,71,72]. Photophobia and its associated migraine-like symptoms are major sources of functional impairment in TBI [73]. Other commonly reported visual symptoms of mTBI include disorders of extraocular movements, affecting saccadic movements and smooth pursuits. Patients that have experienced TBI exhibit latencies such as lagged smooth pursuit movements as well as position errors and reduced acceleration in saccadic movements [74]. Difficulties with reading in TBI patients are noteworthy, with documented abnormalities including increased fixations and regressions per 100 words, reduced reading rates, and lower comprehension and sophistication in reading level [75].

## 7. Visual Pathway, Parietal Lobes and Vision

**Figure 2:**
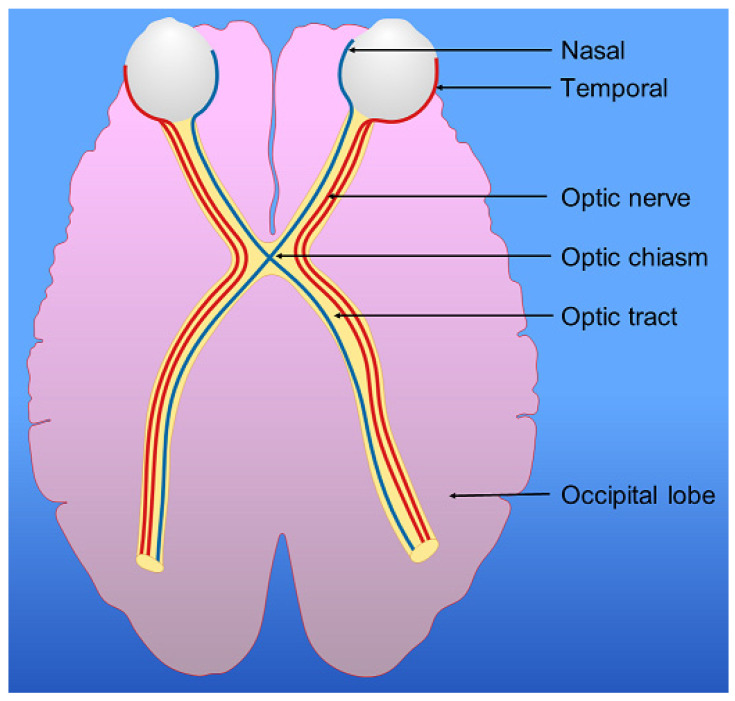
The visual pathway. The optic nerves from each eye partially cross at the optic chiasm so that fibers from the nasal half of each retina cross over to the contralateral optic tract. Fibers from the temporal portion of each retina remain ipsilateral. As a result, the left optic tract contains fibers originating from the left temporal retina, and the right nasal retina while the right optic tract contains fibers originating from the right temporal retina, and the left nasal retina.

Optic nerves from each eye transport visual impulses from retinal ganglion cells in the retina to the optic chiasm and then to higher visual processing centers in the brain. As a result of partial decussation at the optic chiasm, each optic tract contains the fibers from the ipsilateral temporal and contralateral nasal retina (Figure 2).

The optic nerve and tracts can be damaged from transmitted forces during TBI, even when the impact is minor, and can result from either the primary or secondary injury. The mechanism of traumatic optic neuropathy is not fully understood, but may result from tension on the nerve or nerve compression and involve damage to the axons and/or reduction of the blood supply to the nerve [76,77,78]. The visual impairment from optic nerve damage in TBI generally occurs at the time of injury and may vary from a deficit in color vision to loss of visual acuity to sudden, complete visual loss [79,80]. Treatment is difficult and may be medical, with high dose systemic corticosteroids or surgical, with decompression of the optic canal, or a combination of surgery and corticosteroids. Observation is also a valid approach because spontaneous visual recovery is well-documented [81,82,83].

When evaluating the consequences of TBI on ophthalmologic function, a critical region of the brain to consider is the parietal cortex. The posterior parietal cortex is a central associative region of the brain and is located in the center of the brain behind the frontal lobes of the brain and in front of the occipital lobes [84]. This structural proximity lends itself to functional connections among the parietal cortex and the temporal visual area, the occipital visual area, and the prefrontal cortex [85]. The parietal lobes have great significance due to their involvement in sensorimotor integration, decision making, spatial navigation, and short term memory [86,87]. The parietal cortex encodes spatial coordinates and is engaged during the planning of reaching toward a target [88]. Surgery affecting the parietal lobes is associated with a risk of the loss of language and visual field deficits [89].

## 8. TBI Affects the Parietal Lobes, Vestibular System and Visual Perception

### 8.1. Parietal Lobes

The parietal lobes are frequently injured in head trauma, leading to deficits in sensorimotor function, memory, and attention span [90,91]. Given that eyesight is one of the core senses integrated in the parietal cortex, it is therefore critical to examine the ophthalmologic manifestations of TBI in the parietal region. The parietal lobes integrate visual data from rapid eye movements (saccadic movements) to direct the mechanistic reaching movements of the hand, allowing the individual to place an object in space and reach out to grasp it [88,92]. However, when the parietal cortex is impaired, depth perception cannot function properly, causing loss of the ability to perceive the spatial layout of the objects surrounding them [93]. Another consequence of parietal lobe dysfunction occurs in patients who lose the ability to shift their spatial attention or distinguish things on their left or their right side, leading to difficulty navigating even simple tasks such as walking across a room [90,94]. Banal tasks are further impacted in TBI patients that have sustained damage to the parietal lobe, as the parietal lobe plays a crucial role in integrating limb movements to produce coordinated actions [84]. The aforementioned challenges greatly decrease quality of life and increase the risks faced in high-danger zones such as work sites and this can then disqualify the TBI sufferer from employment.

Further manifestations of parietal lobe damage in TBI patients can be observed as functional deficits in speech and language, or as behavioral changes in impulse control and decision-making situations. As far as risk aversion, one study showed that TBI in a rat model chronically altered the propensity of the rats to make high-risk decisions, leading to an increase in risk-taking behavior [95]. Humans with TBI-inflicted damage to the parietal lobes have been observed to experience a decrease in both altruistic behavior and goal-directed behavior, especially when damage is sustained in the lateral parietal cortex [96] This has grave implications on a patient’s quality of life, as apathetic behavior increases the difficulty a patient will have completing everyday tasks [97].

As detailed above, the effects of parietal lobe damage on ophthalmologic function are complex phenomena that are often experienced as confusing by the patient. This complicates the process of detecting visual abnormalities in TBI patients, since many of the described deficits are not detectable with standard eye examinations. Detecting parietal lobe damage via ophthalmologic evaluation includes an exhaustive evaluation of visual field measurement to detect abnormalities in depth perception [98,99]. Abnormalities in depth perception are often difficult for patients to describe. Therefore, evaluation with specialized equipment is key to diagnosing a patient and providing them with accurate medical care. This equipment also allows for the assessment of a patient’s peripheral vision, which is also often affected in patients with parietal lobe damage. Accurately detecting these visual abnormalities are essential to determining the long-term consequences a patient will experience after sustaining TBI, as visual dysfunction can alter the ability of a patient to work or live independently.

Another common ophthalmologic manifestation of TBI is homonymous quadrantanopia, a type of incomplete homonymous hemianopia that results from lesions occurring in the postchiasmal visual pathways in the occipital or parietal lobe [100,101,102]. Quadrantanopia is loss of a visual field quadrant and homonymous quadrantanopia involves the loss of the congruent quadrant on the same side in both eyes [103]. It is commonly known as the “pie in the sky” phenomenon and causes difficulty in visual scanning and detection, leading to complications in everyday life processes such as driving a car or operating other machinery [104,105]. Although some spontaneous recovery may occur, rehabilitation generally involves visual aids and strategies to compensate for the loss [106,107,108].

Injury to the primary visual pathways through the parietal and temporal lobes should be understood separately from disorders of higher-level visual processing. Since damage occurs before transfer to the occipital lobe for primary processing, the homonymous hemianopsias are very distinct and well-defined on visual field testing [109].

### 8.2. Vestibular System

Deficits in multisensory processing in TBI patients can also be caused by vestibular impairment. The vestibular system, located inside the ear, is essential for the integration of sensory information to produce balanced body and eye movements for posture control and navigation [110]. Visual tracking neurons receive vestibular information in cortical sites located in the associative parietal and temporal cortex. Parietal regions are involved in processing vestibular information for the perception of self-motion [111]. In cases of post-concussion or mTBI vestibular dysfunction, vestibular impairment causes a myriad of symptoms related to imbalance and disorientation, which often manifests as dizziness/vertigo and/or lightheadedness and may be accompanied by nausea as well [112,113]. For non-hospitalized TBI patients, vestibular function testing detects abnormalities in somewhere between about 30 to 60% of those tested and symptoms may continue for in excess of one year in 10 to 15% of patients with a mild concussion [10,114,115,116,117]. Vestibular symptoms make return-to-work difficult and interfere with many normal activities. Symptoms are most prominent with head movement or when the patient is moving on foot [118].

Reciprocal interactions occur between visual and vestibular cortical regions. Altered vestibular function has been hypothesized to cause postconcussive visual motion sensitivity. Allen et al. found that post-concussion patients with subacute vestibular impairment symptoms showed exaggerated activation in the multisensory processing centers involved in visual-vestibular sensory processing [119]. They hypothesize that these patients developed overreliance on visual stimuli to compensate for vestibular impairment and that this may lead to difficulty in recovering after TBI. Targeted vestibular rehabilitation may help in these cases [120].

### 8.3. Visuospatial Neglect

Visuospatial neglect occurs when spatial awareness is lost on the side opposite the injured hemisphere. Patients display reduced attention unilaterally and will not explore spontaneously or respond to stimuli originating in the contralesional hemi-space [121]. Visuospatial neglect happens more often when the right parietal lobe is injured resulting in left hemi-field inattention [122]. It is most commonly seen after hemispheric stroke, but may occur after TBI as well and is associated with poorer recovery. It leads to postural instability and the risk of falls. Treatment is usually partial visual occlusion via eye-patching of the non-neglected half of the visual spatial field [123]. Prism adaptation, which laterally displaces the visual field using special lenses, can improve the performance of spatial tasks [124].

## 9. TBI, Insomnia, and the Eyes

The incidence of sleep disruption in patients that have experienced significant head trauma has been reported in the range of 27% to 72.7% in several studies, depending on the type of sleep disruption and the severity of TBI [125,126]. Commonly observed sleep disruptions in TBI patients include hypersomnia, insomnia and daytime sleepiness [127,128,129]. This heightened prevalence in abnormal sleep patterns in TBI patients is present in both patients who have a history of sleep issues and patients who have never had sleep difficulties and is very troubling to patients [130,131]. Sleep problems can exacerbate depression, stress and pain [132,133,134]. Lack of sleep also makes sufferers more accident-prone [135].

Disruptions in sleep patterns impede the rehabilitation process, as they result in lethargy, attentional deficits, and the overall impairment of cognitive function that significantly reduces quality of life [136,137]. Sleep deficits interfere with brain recovery pathways that occur during healthy sleep cycles. Patients that experience sleep-wake cycle disturbances display abnormal levels of neurotransmitters, leading to irregular neuronal activation patterns and the disruption of brain repair mechanisms [137].

The chronic lack of sleep observed in TBI patients has damaging effects on recovering eye cells [138]. The cells in the retina are metabolically active for the entire day as they process information from the electromagnetic waves they are continuously receiving [139]. Altered circadian rhythmic function has been linked to imbalances in metabolic homeostasis, causing abnormal regulation of gene transcription and the dysfunction of glucose metabolism [140]. Therefore, sleep disruption inhibits the ability of retinal cells to replenish their energy reserves and leads to issues in homeostatic maintenance in cells such as the rods and cones. This cellular homeostatic imbalance in the rods and cones causes wear down in the main photoreceptors of the eye, resulting in visual fatigue and hindering recovery.

Another type of sleep disorder that has been found in TBI patients is sleep apnea, in which a patient experiences irregular breathing patterns during sleep [141]. In addition to the sleep deficits caused by sleep apnea, the repeated cessation of breathing in sleep apnea patients can amplify the neurological sequelae of TBI, as it causes hypoxemia and is negatively correlated with cognitive function in TBI patients [142].

## 10. The Ruptured Globe

The eyeball itself can be harmed directly via blunt or sharp trauma. In cases where direct damage occurs to the eyeball, it can be difficult to address the injury early in the emergency room assessment, as the eyelids tend to be highly swollen and bruised after impact. This may result in the failure to identify an injury to the eyes, as doctors may be hesitant to forcibly separate the upper and lower eyelids of the trauma patient in order to inspect the eyeball.

A serious eye trauma-related injury not to be overlooked is a ruptured globe. A ruptured globe or open globe injury is a full-thickness injury to the cornea, sclera or both of these components of the protective shell of the eyeball [143,144]. It can be caused by a blunt or penetrating force and exposes the fragile contents of the interior of the eye, which are easily perturbed [145]. These highly critical tissues are extremely sensitive to any element of disruption, increasing the probability of vision loss in patients. The ruptured globe must be recognized immediately as an ophthalmologic emergency and surgical repair should be undertaken as soon as possible. Ophthalmic examination must be attempted on patients with a ruptured globe as preoperative visual acuity is an important prognostic indicator for surgical outcome [146]. The presence of any intraocular foreign bodies can be detected by examination or CT scan [147,148,149]. Since time elapsed between injury and surgery is another predictor of outcome, ruptured globe surgery usually takes place within 12 to 24 h after trauma to restore and preserve the structural integrity of the eyeball.

The prognosis of ruptured globe depends heavily upon the size and severity of the rupture [143,144]. A small laceration to the cornea tends to have a better prognostic outcome than a deeper rupture, with a higher probability of vision recovery due to the cornea’s location on the surface of the eye. Larger lacerations or ruptures of the more posterior sclera, often cause damage to the retina and carry a much poorer prognosis due to the immense difficulty of correcting deep eye damage. In the most severe of cases, patients may present with no light perception and the eye may not be salvageable. Surgery on patients with no light perception often ends in enucleation, or the complete removal of the eye, to prevent sympathetic ophthalmia, a sight-threatening disorder caused by trauma to the contralateral eye [150]. However, surgical globe repair may be a preferred alternative [151,152,153].

Permanent vision loss is a severe complication of head and facial trauma. Prompt treatment of the ruptured globe is important to avoid permanent loss of vision. The initial surgical effort in severe cases is to restore the integrity of the globe and prevent leakage of internal ocular tissues outside the eyeball. This is to prevent secondary damage to the structural integrity of the eyeball Future surgeries are often required to remove blood, fix the retina, and remove damaged lenses or traumatic cataracts. If these efforts are unsuccessful, it may become necessary to remove the eye entirely via enucleation.

## 11. An Ophthalmologist Clinical Perspective

### 11.1. Diagnostic Tools for Ophthalmologic Evaluation of TBI

Oculomotor assessment following TBI generally includes an evaluation of smooth pursuit eye movements and fixation, vergence and accommodation and saccades [154]. Poorer oculomotor function is correlated with more symptoms post-TBI and difficulties with everyday activities [155,156].

Visual field defects are frequently observed after TBI [157]. Formal visual field testing is generally performed in an ophthalmology outpatient setting. The cost of these devices limits acquisition in more general medical practices [158].

The King Devick test, which can be routinely performed in an ophthalmology office or on the sideline during sporting events, is a widely used standardized test to assess for TBI by evaluating cognitive processing speed and rapid shifting of gaze [159]. This is a simple yet sensitive test based on the ability to complete a rapid number-naming task on an iPad or paper [160]. It is not sufficient to use this test alone and it is known to have a high false-positive rate [161].

The Brain Injury Vision Symptom Survey (BIVSS), a 28-item symptom questionnaire, is useful in documenting vision complaints and distinguishing between patients with and without mild-to-moderate TBI [108,160,162]. This test is considered a valid instrument for vision symptoms that can be administered online, in-person or by healthcare providers.

### 11.2. Visual Consequences of TBI in Daily Life

The undesirable consequences of TBI are experienced by in excess of 5 million people spanning all ages in the United States alone [8]. The vast majority of TBI falls into the mild category and those affected may never be seen by a healthcare provider and never receive a formal diagnosis [163]. Some may enter an emergency department or urgent care facility immediately after an automobile collision or other type of accident and then be discharged with no follow-up. Routine CT and MRI scans of the brain are somewhat insensitive measures of mild TBI and often yield a normal reading. Unfortunately, despite normal imaging, patients may remain symptomatic months to years after injury [164]. Even if these individuals receive a comprehensive neurologic evaluation, effective interventions are lacking.

Although visual symptoms and findings are common, they may go unnoticed by medical providers. The importance of ophthalmologic evaluation of such patients is underestimated. The enormous role of the brain in visual information processing is not subsumed within a standard eye exam [165,166]. There is a need for coordination among specialists, including ophthalmologists, in order to address TBI-related losses in ability to gather and process huge amounts of visual data, processing of which is essential for everyday life [167,168].

Reading text on a computer screen has become one of the single most important tasks in an ever-evolving technological world and fatigue and eyestrain result from this common task [169,170]. For many TBI patients this fatigue becomes burdensome and disabling. Many individuals rely upon a smart phone, a powerful mini-computer that has become a necessary tool to multitudes around the globe. The tiny letters and numbers on various screens are reported to appear blurry or hard to read after TBI [171]. Driving is essential to independence for some and the light sensitivity so often experienced by TBI patients may interfere with this activity and act as a dangerous distraction [172]. Sleep disruption induced by mild TBI can exacerbate visual disturbances and impair perception [171,172,173,174].

The treatment of the visual symptoms and findings of TBI involve the prescription of eyeglasses with tints and prism combinations, bi-nasal occlusion as well as light-filtering lenses [175,176,177]. Interdisciplinary care is needed to get the best outcomes of rehabilitation for these patients [178].

## 12. Conclusions

This review has explored and discussed the pathophysiology and difficulty in the diagnosis of mild-to-moderate TBI. The various neuroradiological modalities employed to determine the extent of the damage and their shortcomings have been covered. Normal MRI, CT, and other test results can be deceptive and conflict with subjective symptomatology. The long-term sequelae of mild-to-moderate TBI are a serious concern with consequences affecting quality-of-life, productivity and the economic viability of patients. Common symptoms include confusion, headache, balance problems, nausea, vomiting, vertigo, visual disturbances, photophobia, fatigue, insomnia, and sound sensitivity. Emotional and behavioral symptoms such as depression, mood swings, agitation and anxiety are also associated with TBI. Achieving functional recovery is difficult and the available rehabilitation options offer modest benefits in many cases. Prolonged effects on visual pathways and visual processing are often prominent and can affect visuo-motor coordination and tasks such as driving, reading and the use of computer technology. Preventing future TBIs is critical because the cumulative number of head injuries suffered negatively impacts recovery. While novel approaches to the treatment of TBI are urgently needed, head injury prevention programs and the implementation of education and outreach where possible can yield tangible results.

## Figures and Tables

**Table 1:** Summary of classifications systems based on imaging for risk stratification and prediction of mortality in TBI.

Classification	Scoring	Key Features
Marshall (1992) [53]	Diffuse Injury I to Diffuse Injury VI	Diffuse injury I—No visible intracranial pathology on CT. Progresses up to Diffuse Injury VI with high or mixed density lesion > 25 mL not surgically evacuated. Evaluates perimesencephalic cisterns, midline shift, and presence of a mass lesion.
Rotterdam (2006) [54]	1 to 6	4 scored elements: basal cistern compression status; degree of midline shift; epidural hematomas, intraventricular and/or subarachnoid hemorrhage. Differentiates between types of mass lesions, recognizes more favorable prognosis for epidural hematomas.
Stockholm (2010) [55]	Traumatic subarachnoid hemorrhage score Range: (0 to 6)	Builds on Marshall and Rotterdam. Adds separate scoring for traumatic subarachnoid hemorrhage. Magnitude of midline shift used as a continuous variable (not dichotomous) for prediction of favorable or unfavorable outcome. Incorporates diffuse axonal injury.
Helsinki (2014) [56]	−3 to 14	Refined to include type of mass lesion (subdural, intracerebral or epidural hematoma. Intraventricular hemorrhage as a predictor of outcome. Includes suprasellar cisterns status (normal, compressed, obliterated).
NeuroImaging Radiological Interpretation System (NIRIS) (2018) [57]	NIRIS 0 to NIRIS 4	Score gives management guidance: NIRIS 0—patients typically discharged, NIRIS 1—follow-up neuroimaging and/or hospital admission, NIRIS 2—admission to an advanced care unit, NIRIS 3—neurosurgical intervention, NIRIS 4—high likelihood of fatal outcome from TBI.

## Data Availability

All data listed in this manuscript is publically available from manuscripts found on PubMed.
